# Visceral Adiposity, Inflammation, and Testosterone Predict Skeletal Muscle Mitochondrial Mass and Activity in Chronic Spinal Cord Injury

**DOI:** 10.3389/fphys.2022.809845

**Published:** 2022-02-10

**Authors:** Jacob A. Goldsmith, Raymond E. Lai, Ryan S. Garten, Qun Chen, Edward J. Lesnefsky, Robert A. Perera, Ashraf S. Gorgey

**Affiliations:** ^1^Spinal Cord Injury and Disorders, Hunter Holmes McGuire Veterans Affairs Medical Center (VAMC), Richmond, VA, United States; ^2^Department of Physical Medicine and Rehabilitation, Virginia Commonwealth University, Richmond, VA, United States; ^3^Department of Kinesiology and Health Sciences, Virginia Commonwealth University, Richmond, VA, United States; ^4^Medical Service, Hunter Holmes McGuire VA Medical Center, Richmond, VA, United States; ^5^Division of Cardiology, Department of Medicine, Pauley Heart Center, Virginia Commonwealth University, Richmond, VA, United States; ^6^Department of Biostatistics, Virginia Commonwealth University, Richmond, VA, United States

**Keywords:** spinal cord injury, visceral adipose tissue, mitochondria, inflammation, growth factors, testosterone

## Abstract

**Background:**

Mitochondrial health is an important predictor of several health-related comorbidities including obesity, type 2 diabetes mellitus, and cardiovascular disease. In persons with spinal cord injury (SCI), mitochondrial health has been linked to several important body composition and metabolic parameters. However, the complex interplay of how mitochondrial health is affected has yet to be determined in this population.

**Objective:**

In this study, we examined the contribution of visceral adiposity, inflammatory biomarkers, testosterone and circulating serum growth factors as predictors of mitochondrial health in persons with chronic SCI.

**Participants:**

Thirty-three individuals with chronic SCI (*n* = 27 Males, *n* = 6 Females, age: 40 ± 13.26 years, level of injury: C4-L1, BMI: 23 ± 5.57) participated in this cross-sectional study.

**Methods:**

Visceral adipose tissue (VAT) was measured via magnetic resonance imaging (MRI). After an overnight fast, serum testosterone, inflammatory biomarkers [interleukin 6 (IL-6), tumor necrosis factor alpha (TNF-α), c-reactive protein (CRP)], and anabolic growth factors [insulin-like growth factor 1 (IGF-1), insulin-like growth factor binding protein 3 (IGFBP-3)] were measured. Skeletal muscle biopsies were obtained from the vastus lateralis muscle to measure citrate synthase (CS) and Complex III activity. Regression analyses were used to examine predictors of mitochondrial mass and activity.

**Results:**

CS activity was negatively associated with VAT (*r*^2^ = 0.360, *p* < 0.001), CRP (*r*^2^ = 0.168, *p* = 0.047), and positively associated with testosterone (*r*^2^ = 0.145, *p* = 0.042). Complex III activity was negatively associated with VAT relative to total lean mass (VAT:TLM) (*r*^2^ = 0.169, *p* = 0.033), trended for CRP (*r*^2^ = 0.142, *p* = 0.069), and positively associated with testosterone (*r*^2^ = 0.224, *p* = 0.010). Multiple regression showed CS activity was significantly associated with VAT + CRP (*r*^2^ = 0.412, *p* = 0.008) and VAT + Testosterone (*r*^2^ = 0.433, *p* = 0.001). Complex III activity was significantly associated with VAT relative to total trunk cross-sectional area (CSA) + CRP (VAT:total trunk CSA + CRP; *r*^2^ = 0.286, *p* = 0.048) and VAT + Testosterone (*r*^2^ = 0.277, *p* = 0.024).

**Conclusion:**

Increased visceral adiposity and associated inflammatory signaling (CRP) along with reduced testosterone levels predict mitochondrial dysfunction following SCI. Specifically, lower VAT_CSA_ and higher testosterone levels or lower VAT_CSA_ and lower CRP levels positively predict mitochondrial mass and enzyme activity in persons with chronic SCI. Future research should investigate the efficacy of diet, exercise, and potentially testosterone replacement therapy on enhancing mitochondrial health in chronic SCI.

**Clinical Trial Registration:**

[www.ClinicalTrials.gov], identifier: [NCT02660073].

## Introduction

Cardiometabolic disorders are a leading cause of mortality among persons with spinal cord injury (SCI) (Schladen and Groah, 2014). Recent guidelines have emerged to highlight the magnitude of the problem and provide assessment tools for researchers and clinicians to distinguish those at risk (Nash and Gater, 2020). Cardiometabolic syndrome presents as a cluster of disorders, including impaired glucose tolerance, insulin resistance, dyslipidemia, central obesity, and elevated blood pressure (Després and Lamarche, 1993; Després et al., 2001; Grundy et al., 2004). Cardiometabolic syndrome impacts more than 50% of persons with SCI (LaVela et al., 2006; Dopier Nelson et al., 2007). Today, the root of the problem remains unresolved and likely to continue without appropriate intervention.

Recent emerging cross-sectional studies have clearly associated central obesity with cardiometabolic disorder after SCI. Earlier studies indicated the link between waist circumference and cardiometabolic diseases in persons with SCI (Buchholz and Bugaresti, 2005; Gill et al., 2020). It appears that waist circumference reflects increased visceral adiposity. Gorgey and Gater (2011) were among the first to show that with increasing visceral adipose tissue (VAT), persons with SCI may suffer from impaired glucose intolerance, insulin resistance, and dyslipidemia. Another study showed that people with SCI had 58% greater VAT than waist circumference matched controls (Edwards et al., 2008). Using a simple linear regression model, Sumrell et al. (2018) showed that a waist circumference greater than 86.5 cm is equivalent to VAT equal to or greater than 100 cm^2^. The same work demonstrated that VAT > 100 cm^2^ was associated with decreased insulin sensitivity, increased inflammatory biomarkers, and reduced oxygen uptake. In a follow-up trial, the same research group showed that a waist circumference of 86.5 cm distinguishes those at risk of developing cardiometabolic disorders in persons with SCI (Gill et al., 2020). A recent review summarized potential mechanisms for increasing VAT to contribute to the prevalence of cardiometabolic diseases after SCI (Goldsmith et al., 2021).

Previous work has alluded to several potential mechanisms that likely trigger VAT-associated cardiometabolic disorders (Seidell et al., 1990; Nicklas et al., 2003; Fox et al., 2007; Katzmarzyk et al., 2013; Lee et al., 2018; Gorgey et al., 2021). Although most studies have not demonstrated causality, these studies established potential mechanisms that warrant further investigation. Farkas et al. (2018) and Farkas and Gater (2018) noted that with increasing VAT, there is a potential increase in inflammatory biomarkers, mainly tumor necrosis factor-alpha (TNF-α) and interleukin-6 (IL-6). Abilmona et al. (2019) noted that, in persons with SCI, hypogonadal individuals (<300 ng/dL) are likely to have greater VAT than persons with normal testosterone levels. Finally, O’Brien et al. (2017b) described an association between increasing VAT and mitochondrial dysfunction in persons with SCI.

Mitochondrial health is an important predictor of several health-related comorbidities, including obesity, type 2 diabetes mellitus and cardiovascular disease (Poznyak et al., 2020). Mitochondrial function is linked to several important body composition and metabolic parameters in persons with SCI. Specifically, mitochondrial mass (citrate synthase activity) and Complex III activity have been negatively associated with increased body fat and reduced cardiometabolic health (O’Brien et al., 2017a,b, 2018). Based on the above evidence, it appears that VAT exerts deleterious cardiometabolic effects in persons with SCI, by increasing systemic inflammation and diminishing testosterone levels that lead to mitochondrial dysfunction. This cross-sectional study aimed to examine the contribution of visceral adiposity, inflammatory biomarkers, testosterone, and circulating serum growth factors as predictors of mitochondrial health in persons with chronic SCI.

## Materials and Methods

### Participants

Thirty-three individuals with chronic SCI (age: 40 ± 13.26 years, level of injury: C5-L1, BMI: 23 ± 5.57) participated in this cross-sectional study (registered at clinicaltrials.gov: NCT02660073). Only cross-sectional baseline data are presented in this manuscript. All procedures were in accordance with the ethical standards of the Helsinki Declaration of 1,964 and its later amendments. The McGuire Veteran Affairs Investigation Research Board and the Virginia Commonwealth University (VCU) Office of Research and Innovation approved the current study. A neurological examination was performed per the International Standards for Neurological Classification of SCI (ISNCSCI) to determine the American Spinal Injury Association (ASIA) Impairment Scale (AIS) for each participant. Participants provided written, informed consent before the study commenced. Participants with the following pre-existing medical conditions were excluded: active urinary tract infection, those using insulin, hematocrit > 50%, stage 3 pressure sore or above, uncontrolled hypertension, cardiovascular disease or uncontrolled type 2 diabetes mellitus, and individuals with neck of femur or total body osteoporosis (T-score ≤ −2.5 according to the World health organization guidelines) (Reginster and Burlet, 2006; Gorgey et al., 2019a). All participants were instructed to abstain from exercise, alcohol, and caffeine consumption 24 h before the examination. Participants underwent a general physical examination to rule out any preexisting cardiac problems that included measuring vital signs and a resting 12-lead electrocardiogram. After a 10–12 h fast, a cannula was inserted into an antecubital vein of one arm for blood sampling. Fasting whole-blood samples were drawn into serum separator and potassium oxalate/sodium fluoride tubes and centrifuged to collect serum and plasma samples, respectively. The majority of blood samples were sent to the Chemistry Pathology Laboratory for analysis; however, a subset was sent to a research lab at VCU for further analysis using the same protocol and assay kits. Inflammatory biomarkers (Tumor necrosis factor-alpha; TNF-α, Interleukin-6; IL-6, and c-reactive protein; CRP) were also analyzed in serum samples by enzyme-linked immunosorbent assays (ELISA) (ALPACO; Salem, NH). Total serum testosterone was measured by liquid chromatography with isotope dilution mass spectrometry detection after supported liquid extraction (Esoterix, Inc.). Testosterone levels in each sample were calculated from a linear plot generated by purified testosterone standards ranging from 2.5 to 5,000 ng/dL. Serum insulin-like growth factor-1 (IGF-I) and insulin-like growth factor-binding protein 3 (IGFBP-3) concentrations were measured with immunoassays (Quantikine R&D Systems, Inc., Minneapolis, MN, United States).

### Magnetic Resonance Imaging

Abdominal MRI scans were imaged using a 1.5- or 3 Tesla magnet (General Electric, Waukesha, WI) whole-body scanner, using a fast spin-echo sequence described previously (Gorgey et al., 2019b). Transverse images (slice thickness of 0.8 cm, inter-slice space of 1.2 cm) were captured from the xiphoid process to the femoral heads. Depending on the individual’s torso length, approximately 20 ± 30 images were obtained. Participants were asked to remain as still as possible during the entirety of the scan. In addition, participants were instructed to hold their breath for approximately 20 s to prevent respiratory artifacts from altering image quality. Images were sequenced anatomically using Image-J software (National Institute of Health, Bethesda, Maryland) and analyzed using Win Vessel software (Win Vessel 2.0, Ronald Meyer, Michigan State University, East Lansing, MI, United States). Each image was automatically segmented into fat and muscle, with bone and background tissue identified based on its signal intensity. Abdominal adipose tissue was separated into subcutaneous adipose tissue (SAT) and VAT depots. An experienced technician manually identified regions of interest guided by anatomical landmarks. The cross-sectional areas (CSA) of these different compartments were used to derive the VAT:SAT ratio to control for regional adiposity. The total area within the outer border of the trunk represented the total trunk CSA, which was used to normalize VAT_CSA_ to Total trunk CSA (VAT:total trunk CSA ratio) (Abilmona and Gorgey, 2018). All values were averaged across images to reflect the whole torso.

### Dual-Energy X-Ray Absorptiometry

Total body and regional DXA scans were performed using a GE Lunar iDXA (Lunar Inc., Madison, WI, United States) bone densitometer at the Hunter Holmes VA Medical Center. All scans were performed and analyzed using Lunar software version 10.5. After scanning, total and regional % fat mass and fat-free mass were determined using DXA software. The longitudinal precision of total and regional body composition using DXA has been determined in persons with SCI (Lester et al., 2019). As previously described, VAT mass was made relative to SAT mass to account for differences in SAT mass between individuals. Additionally, VAT was made relative to total lean mass (TLM) and trunk CSA to account for differences between individuals, as previous research has demonstrated a relationship between VAT mass, total trunk mass, and LM (O’Brien et al., 2017b; Abilmona et al., 2019).

### Enzyme Activities

Muscle biopsies from the vastus lateralis of the right leg were collected using a 14-gauge Tru Cut™ needle under local anesthesia (2% lidocaine). Samples were snap-frozen in liquid nitrogen and stored at −70°C. Connective and adipose tissue was removed from a portion of the sample (∼10–25 mg), then homogenized in ice-cold buffer containing 220 mM mannitol, 70 mM sucrose, 5 mM MOPS, 2 mM EDTA, with cOmplete™ protease inhibitor cocktail, pH 7.4 (Sigma-Aldrich). The homogenate was centrifuged at 2,000 rpm (371 g) for 5 min at 4°C and the supernatant was used for analysis. Protein concentration was quantified, and samples were solubilized in 1% potassium cholate. Homogenization and assays were completed on the same day. CS and Complex III activity were measured spectrophotometrically in duplicate or triplicate as previously described (Brass et al., 2001; O’Brien et al., 2017a). CS activity, a measure of mitochondrial mass, was measured as the formation of thionitrobenzoate at 412 nm after the addition of 5, 5-dithiobis-(2, 4-nitrobenzoic acid), acetyl-CoA, and oxaloacetate (*n* = 32). Complex III activity was reflected by the rate of cytochrome c reduction in absorbance at 550 nm (*n* = 32) (Brass et al., 2001; Spinazzi et al., 2012). Antimycin A was used to inhibit Complex III. The activity of Complex III was expressed as the antimycin A-sensitive rate. Absorbance was measured before and after the addition of oxaloacetate, and background absorbance was subtracted from the final reading. Data were converted from arbitrary units per minute to nmol/min by using the extinction coefficients of 13.6 mM^–1^ cm^–1^ for CS and 19.1 mM^–1^ cm^–1^ for Complex III. Data were normalized to mg of protein added.

### Statistical Analysis

All data were analyzed for normality of distribution. Data that were not normally distributed were log-transformed to permit the use of parametric statistics. Single and multiple linear regression models were used to examine the relationships between measures of mitochondrial mass (citrate synthase activity) and activity (Complex III), visceral adiposity, serum inflammatory biomarkers, anabolic growth factors, and testosterone. We used a maximum of two predictors in each regression to avoid multicollinearity issues due to the relatively low sample size. Instead of adding variables including time since injury (TSI), level of injury (LOI), and age as predictors in these models, we controlled these variables using weighted least squares regressions. We continued with multiple regression models only if a predictor was independently significant with either CS or Complex III activity. Statistical analyses were performed using SPSS (SPSS Statistics version 24, IBM Corp., Armonk, United States). Statistical significance was accepted at *a priori* of α ≤ 0.05.

## Results

### Participant Characteristics

**FIGURE 1:**
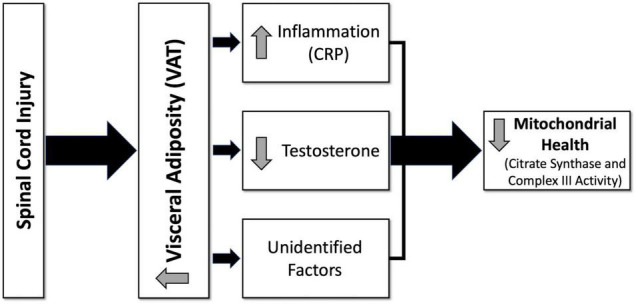
Hypothesized diagram of factors predicting mitochondrial health following spinal cord injury (SCI). After SCI, visceral adiposity increases and releases inflammatory cytokines that negatively affect testosterone levels and mitochondrial health. Visceral adiposity, inflammation, and testosterone are involved in this deleterious process; however, there are still factors that remain unidentified. CRP, c-reactive protein; VAT, visceral adipose tissue.

Participant demographics and injury characteristics are presented in Table 1. Twenty-two were paraplegic (T1-L1) and 11 were tetraplegic (C4-C8). Participants ranged in age from 20 to 61 and BMI ranged from 14.2 to 35.3 kg/m^2^. Age, height, weight, BMI, and TSI were not significantly different between tetraplegics and paraplegics or Caucasians and African Americans. Inflammatory biomarkers, anabolic growth factors, serum testosterone levels, mitochondrial enzyme activity, and MRI outcomes are presented in Table 2. Values were not significantly different between paraplegics and tetraplegics or Caucasians and African Americans. Central adiposity was apparent in 21 and 32% of the participants using cutoffs of ≥ 100 cm^2^ MRI VAT_CSA_, and ≥ 0.66 VAT:SAT ratio, respectively (Gorgey et al., 2011, 2014). Importantly, a fraction of the samples (eight samples used for IL-6, TNF-α, IGF-1, and IGFBP-3) sent to VCU for further analysis experienced degradation that resulted in erroneous values. Therefore, we excluded these samples from analyses, which resulted in an uneven sample size depending on the marker be analyzed (Table 2). Figure 1 shows the hypothesized factors predicting mitochondrial health following SCI. This hypothesized model shows that, after SCI, visceral adiposity increases and releases inflammatory cytokines that negatively affect testosterone levels and mitochondrial health. While it is clear that visceral adiposity, inflammation, and testosterone are involved in this deleterious process, there are still factors that remain unidentified.

**TABLE 1:** Baseline demographics and spinal cord injury characteristics for 33 participants.

Ethnicity	Caucasian: *n* = 18
	African American: *n* = 15
Sex	Male: *n* = 27 Female: *n* = 6
Age (year)	40 ± 13.00
Weight (kg)	70 ± 15.18
Height (cm)	175 ± 8.99
BMI (kg/m^2^)	23 ± 5.25
Level of injury (range)	C5–L1
Time since injury (yr)	11 ± 10.59
AIS (score)	A: *n* = 19
	B: *n* = 9
	C: *n* = 5
SCI classification	Paraplegia: *n* = 22
	Tetraplegia: *n* = 11

*BMI, body mass index; AIS, American Spinal Injury Association Impairment Scale; AIS-A, complete motor and sensory loss below the level of injury; AIS-B, complete motor loss and incomplete sensory loss below the level of injury; AIS-C, incomplete motor and sensory loss with less than half of the muscles tested below the LOI graded ≥ 3. Mean ± SD unless otherwise noted.*

**TABLE 2:** Citrate synthase activity, inflammatory biomarkers, anabolic growth factors, and serum testosterone levels.

Anabolic growth factors	Mean ± SD	Range	Sample size
IGF-1 (ng/mL)	143.99 ± 58.73	72.90–267.85	25
IGFBP-3 (ng/mL)	1851.40 ± 368.57	1320.50–2598.55	25
**MRI and DXA measures**			
Total trunk_CSA_ (cm^2^)	571.44 ± 174.17	323.16–940.64	28
SAT_CSA_ (cm^2^)	136.43 ± 100.30	22.97–369.89	28
VAT_CSA_ (cm^2^)	71.73 ± 64.97	4.06–220.01	28
VAT:SAT ratio	0.64 ± 0.47	0.10–2.22	28
VAT:total trunk CSA	0.11 ± 0.08	0.01–0.30	28
VAT:TLM	0.0015 ± 0.0013	0.0001–0.0043	28
VAT:Leg LM	0.0054 ± 0.0049	0.0004–0.02	28
TLM (kg)	448.48 ± 77.57	288.83–616.34	32
Leg LM (kg)	131.69 ± 28.58	64.55–188.77	32
**Inflammatory biomarkers**			
CRP (ng/mL)	14580.81 ± 19705.45	185.0–73530.25	25
IL-6 (pg/mL)	3.18 ± 2.49	0.68–9.27	22
TNF-α (pg/mL)	22.50 ± 3.96	15.24–30.63	25
**Serum testosterone levels**			
Testosterone (ng/dL)	346.72 ± 223.71	8.30–751.00	30
**Enzyme activity**			
CS (nmol/mg/min)	101.22 ± 59.83	24.00–303.00	32
Complex III (nmol/mg/min)	190.00 ± 125.51	47.10–679.00	32

*MRI, magnetic resonance imaging; DXA, Dual energy x-ray absorptiometry; SAT, subcutaneous adipose tissue; VAT, visceral adipose tissue; ng/dL, IGF-1, insulin-like growth factor 1; IGFBP-3, insulin-like growth factor binding protein 3; CRP, c-reactive protein; IL-6, interleukin 6; CS, citrate synthase; TNF-α, tumor necrosis factor alpha; nanograms per deciliter; CSA, cross sectional area; Kg, kilograms. Mean ± SD unless otherwise noted.*

### Independent Predictors of Citrate Synthase Activity

**FIGURE 2:**
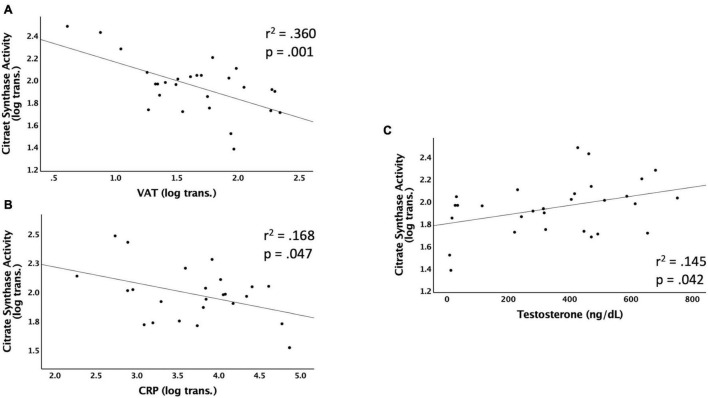
Linear regressions predicting Citrate Synthase (CS) activity. Data that were not normally distributed were log-transformed to permit the use of parametric statistics. The log transformed values of CS enzyme activity are plotted on the Y axis against the predicted values for each variable on the X axis. **(A)** VAT (log transformed) as a predictor of CS activity, **(B)** CRP (log transformed) as a predictor of CS activity, **(C)** testosterone (ng/dL) as a predictor of CS activity. VAT, visceral adipose tissue; CRP, c-reactive protein; ng/dL, nanograms per deciliter.

Figure 2 shows the significant relationships predicting Citrate Synthase (CS) activity. CS activity was negatively associated with VAT (*r*^2^ = 0.360, *p* < 0.001), CRP (*r*^2^ = 0.168, *p* = 0.047), VAT:total trunk CSA (*r*^2^ = 0.372, *p* < 0.001), and VAT:TLM (*r*^2^ = 0.434, *p* < 0.001), and positively associated with testosterone (*r*^2^ = 0.145, *p* = 0.042) (Table 3). CS activity was not independently associated with VAT:SAT, IL-6, TNF-α, IGF-1, or IGFBP-3 (data not shown). The relationships between CS activity and VAT, VAT:total trunk CSA, and VAT:TLM remained significant when controlling for TSI, age, and LOI (Table 4). However, testosterone (*r*^2^ = 0.087, *p* = 0.119) and CRP (*r*^2^ = 0.081, *p* = 0.176) were no longer significant when controlling for TSI. When controlling for age or LOI (paraplegic vs. tetraplegic), CRP (*r*^2^ = 0.249, *p* = 0.013) and testosterone (*r*^2^ = 0.170, *p* = 0.026) were negatively and positively significant, respectively. Moreover, IL-6 showed a significant negative association with CS activity when controlling for age (*r*^2^ = 0.237, *p* = 0.019). IL-6 was only a significant predictor of CS activity in participants over 40 (*r*^2^ = 0.466, *p* = 0.014) but not participants under 40 (*r*^2^ = 0.068, *p* = 0.437).

**TABLE 3:** Single and multiple regressions predicting citrate synthase activity.

Predictor variables	β	*r* ^2^	*p*-value
VAT	−0.600	0.360	<0.001
Testosterone	0.380	0.145	0.042
CRP	−0.410	0.168	0.047
VAT:total trunk CSA	−0.610	0.372	<0.001
VAT:TLM	−0.659	0.434	<0.001
VAT + CRP	−0.530, −0.194	0.412	0.008
VAT + Testosterone	−0.540, 0.272	0.433	0.001
VAT:total trunk CSA + CRP	−0.561, −0.211	0.454	0.004
VAT:total trunk CSA + Testosterone	−0.562, 0.315	0.467	<0.001
VAT:TLM + CRP	−0.579, −0.151	0.444	0.005
VAT:TLM + Testosterone	−0.592, 0.210	0.473	<0.001

*Single and multiple regressions predicting Citrate Synthase activity. Non-significant r^2^ are not shown (non-significant = ns), but non-significant yet trending r^2^ are included. Standardized Beta weights are presented to demonstrate directionality of associations.*

*TSI, time since injury; LOI, level of injury; VAT, visceral adipose tissue; TEST, testosterone; CRP, c-reactive protein; IL-6, interleukin 6; LM, lean mass; TLM, total lean mass; CSA, cross sectional area.*

**TABLE 4:** Single and multiple regressions predicting Citrate Synthase activity after controlling for TSI, LOI, or age.

	Citrate synthase activity *r*^2^ (*p*-value)					
Marker	TSI	β (TSI)	LOI	β (LOI)	Age	β (Age)
VAT	0.174 (0.030)	−0.418	0.432 (<0.001)	−0.657	0.277 (0.005)	−0.526
Testosterone	ns	0.296	0.170 (0.026)	0.412	0.176 (0.023)	0.420
CRP	ns	−0.285	0.249 (0.013)	−0.499	0.184 (0.036)	−0.429
VAT:total trunk CSA	0.162 (0.038)	−0.402	0.435 (0.001)	−0.660	0.285 (0.004)	−0.534
VAT:TLM	0.264 (0.006)	−0.514	0.507 (0.001)	−0.712	0.376 (0.001)	−0.613
VAT + CRP	0.294 (0.044)	−0.479, −0.216	0.503 (0.002)	−0.546, −0.251	0.365 (0.017)	−0.449, −0.273
VAT + Testosterone	0.226 (0.053)	−0.370, 0.238	0.508 (0.001)	−0.592, 0.279	0.382 (0.004)	−0.456, 0.334
VAT:total trunk CSA + CRP	0.245 (0.022)	−0.529, −0.287	0.537 (0.001)	−0.571, −0.263	0.415 (0.008)	−0.494, −0.298
VAT:total trunk CSA + Testosterone	0.250 (0.037)	−0.396, 0.306	0.534 (0.001)	−0.606, 0.322	0.414 (0.002)	−0.483, 0.374
VAT:TLM + CRP	0.343 (0.023)	−0.533, −0.171	0.531 (0.001)	−0.599, −0.200	0.410 (0.009)	−0.515, −0.222
VAT:TLM + Testosterone	0.288 (0.020)	−0.460, 0.176	0.548 (0.001)	−0.642, 0.216	0.432 (0.001)	−0.525, 0.265
IL-6	ns	−0.382	ns	−0.389	0.237 (0.019)	−0.486
VAT + IL-6	ns	−0.479, −0.399	ns	−0.492, −0.326	0.412 (0.011)	−0.419, −0.457

*Weighted least squares regressions predicting Citrate Synthase activity when controlling for TSI, LOI, or age. Non-significant r^2^ are not shown (non-significant = ns), but non-significant yet trending r^2^ are included. Standardized Beta weights are presented to demonstrate directionality of associations.*

*TSI, time since injury; LOI, level of injury; VAT, visceral adipose tissue; CRP, c-reactive protein; IL-6, interleukin 6; LM, lean mass; TLM, total lean mass; CSA, cross sectional area.*

### Multiple Regressions to Predict Citrate Synthase Activity

CS activity was significantly associated with VAT + CRP (*r*^2^ = 0.412, *p* = 0.008) and VAT + testosterone (*r*^2^ = 0.433, *p* = 0.001). Within this model, individuals with lower VAT_CSA_ and lower CRP levels had higher CS activity. Furthermore, individuals with lower VAT_CSA_ and higher testosterone levels had higher CS activity. These relationships remained significant when VAT was made relative to total trunk CSA (CRP: *r*^2^ = 0.454, *p* = 0.004, testosterone: *r*^2^ = 0.467, *p* < 0.001), and TLM (CRP: *r*^2^ = 0.444, *p* = 0.005; testosterone: *r*^2^ = 0.473, *p* < 0.001) (Table 3). These relationships also remained significant when controlling for age [VAT + CRP (*r*^2^ = 0.365, *p* = 0.017), VAT + testosterone (*r*^2^ = 0.382, *p* = 0.004)]. As previously stated, IL-6 became significantly negatively associated with CS activity when controlling for age, and VAT + IL-6 (*r*^2^ = 0.412, *p* = 0.011) was also significantly negatively associated when controlling for age. Specifically, VAT + IL-6 was a significant predictor of CS activity in participants over 40 (*r*^2^ = 0.511, *p* = 0.041) but not in individuals under 40 (*r*^2^ = 0.293, *p* = 0.353). Multiple regressions (VAT + CRP, VAT:total trunk CSA + CRP, VAT:TLM + CRP, VAT:total trunk CSA + testosterone, and VAT:TLM + testosterone), remained significant when controlling for TSI, while VAT + testosterone trended toward significance (*p* = 0.053) (Table 4). When controlling for LOI, VAT + CRP (*r*^2^ = 0.503, *p* = 0.002), VAT + testosterone (*r*^2^ = 0.508, *p* < 0.001), VAT:total trunk CSA + CRP (*r*^2^ = 0.537, *p* = 0.001), VAT:total trunk CSA + testosterone (*r*^2^ = 0.534, *p* = < 0.001), VAT:TLM + CRP (*r*^2^ = 0.531, *p* = 0.001), and VAT:TLM + testosterone (*r*^2^ = 0.548, *p* < 0.001) remained significant (Table 4). All other possible models to predict CS activity were tested and not significant.

### Independent Predictors of Complex III Activity

**FIGURE 3:**
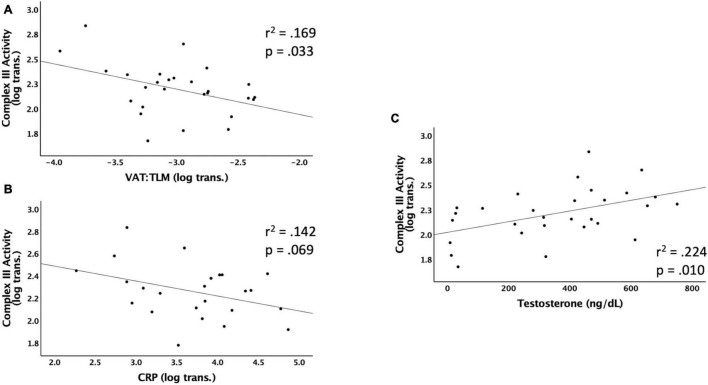
Linear regressions predicting Complex III activity. Data that were not normally distributed were log-transformed to permit the use of parametric statistics. The log transformed values of Complex III enzyme activity are plotted on the Y axis against the predicted values for each variable on the X axis. **(A)** VAT (log transformed) relative to TLM as a predictor of Complex III activity, **(B)** CRP (log transformed) as a predictor of Complex III activity, **(C)** testosterone (ng/dL) as a predictor of Complex III activity. VAT, visceral adipose tissue; CRP, c-reactive protein; TLM, total lean mass; ng/dL, nanograms per deciliter.

Figure 3 shows the significant relationships predicting Complex III activity. Complex III activity was negatively associated with VAT:TLM (*r*^2^ = 0.169, *p* = 0.033) but positively associated with testosterone (*r*^2^ = 0.224, *p* = 0.010). Complex III trended toward significance and was negatively associated with VAT (*r*^2^ = 0.134, *p* = 0.061), CRP (*r*^2^ = 0.142, *p* = 0.069), and VAT:total trunk CSA (*r*^2^ = 0.140, *p* = 0.055) (Table 5). Complex III activity was not independently associated with VAT:SAT, IL-6, TNF-α, IGF-1, or IGFBP-3 (data not shown). Testosterone (*r*^2^ = 0.177, *p* = 0.023) and VAT:TLM (*r*^2^ = 0.210, *p* = 0.016) remained significant when controlling for TSI, but CRP became insignificant. Testosterone (*r*^2^ = 0.269, *p* = 0.004), IL-6 (*r*^2^ = 0.195, *p* = 0.035), and VAT:TLM (*r*^2^ = 0.148, *p* = 0.047) were significant when controlling for age, while VAT and VAT:total trunk CSA were no longer significantly associated with Complex III activity (*p* > 0.05; Table 6). All predictors remained significantly associated with Complex III activity when controlling for LOI, while VAT (*r*^2^ = 0.245, *p* = 0.009), CRP (*r*^2^ = 0.204, *p* = 0.027), and VAT:total trunk CSA (*r*^2^ = 0.239, *p* = 0.010) became significant when controlling for LOI (Table 6).

**TABLE 5:** Single and multiple regressions predicting Complex III activity.

Predictor variables	β	*r* ^2^	*p*-value
VAT	−0.366	0.134	0.061
Testosterone	0.473	0.224	0.010
CRP	−0.377	0.142	0.069
VAT:total trunk CSA	−0.374	0.140	0.055
VAT:TLM	−0.411	0.169	0.033
VAT + CRP	−0.315, −0.294	0.270	0.059
VAT + Testosterone	−0.275, 0.390	0.277	0.024
VAT:total trunk CSA + CRP	−0.335, −0.304	0.286	0.048
VAT:total trunk CSA + Testosterone	−0.316, 0.408	0.303	0.016
VAT:TLM + CRP	−0.332, −0.275	0.275	0.055
VAT:TLM + Testosterone	−0.299, 0.359	0.286	0.021

*Single and multiple regressions predicting Complex III activity. Non-significant r^2^ are not reported, but non-significant yet trending r^2^ are included. Standardized Beta weights are presented to demonstrate directionality of associations.*

*TSI, time since injury; LOI, level of injury; VAT, visceral adipose tissue; CRP, c-reactive protein; IL-6, interleukin 6; LM, lean mass; TLM, total lean mass; CSA, cross sectional area.*

**TABLE 6:** Single and multiple regressions predicting Complex III Activity after controlling for TSI, LOI, or age.

	Complex III Activity *r*^2^ (*p*-value)					
Marker	TSI	β (TSI)	LOI	β (LOI)	Age	β (Age)
VAT	0.142 (0.053)	−0.377	0.245 (0.009)	−0.495	ns	−0.315
Testosterone	0.177 (0.023)	0.421	0.254 (0.005)	0.504	0.269 (0.004)	0.519
CRP	ns	−0.262	0.204 (0.027)	−0.451	0.159 (0.053)	−0.399
VAT:total trunk CSA	0.120 (0.077)	−0.346	0.239 (0.010)	−0.488	ns	−0.321
VAT:TLM	0.210 (0.016)	−0.459	0.283 (0.004)	−0.532	0.148 (0.047)	−0.385
VAT + CRP	0.286 (0.048)	−0.459, −0.238	0.371 (0.015)	−0.390, −0.308	0.264 (0.063)	−0.289, −0.334
VAT + Testosterone	0.264 (0.029)	−0.313, 0.355	0.392 (0.003)	−0.401, 0.394	0.300 (0.016)	−0.220, 0.458
VAT:total trunk CSA + CRP	0.317 (0.032)	−0.491, −0.305	0.379 (0.014)	−0.392, −0.324	0.290 (0.046)	−0.326, −0.347
VAT:total trunk CSA + Testosterone	0.291 (0.019)	−0.348, 0.412	0.412 (0.002)	−0.421, 0.422	0.326 (0.011)	−0.270, 0.474
VAT:TLM + CRP	0.316 (0.033)	−0.496, −0.198	0.372 (0.015)	−0.405, −0.284	0.279 (0.053)	−0.323, −0.304
VAT:TLM + Testosterone	0.299 (0.017)	−0.375, 0.307	0.397 (0.003)	−0.418, 0.357	0.313 (0.013)	−0.255, 0.425
IL−6	ns	−0.396	ns	−0.269	0.195 (0.035)	−0.422
VAT + IL−6	ns	−0.468, −398	ns	−0.423, −0.211	0.290 (0.054)	−0.312, −0.418

*Weighted least squares regressions predicting Complex III activity when controlling for TSI, LOI, or age. Non-significant r^2^ are not shown (non-significant = ns), but non-significant yet trending r^2^ are included. Standardized Beta weights are presented to demonstrate directionality of associations.*

*TSI, time since injury; LOI, level of injury; VAT, visceral adipose tissue; CRP, c-reactive protein; IL-6, interleukin 6; LM, lean mass; TLM, total lean mass; CSA, cross sectional area.*

### Multiple Regressions to Predict Complex III Activity

Complex III was negatively associated with VAT:total trunk CSA + CRP (*r*^2^ = 0.286, *p* = 0.048) and trended toward significance for VAT + CRP (*r*^2^ = 0.270, *p* = 0.059) and VAT:TLM + CRP (*r*^2^ = 0.275, *p* = 0.055). Within this model, individuals with lower VAT_CSA_ and lower CRP levels had higher Complex III activity. Complex III was also significantly associated with VAT + testosterone (*r*^2^ = 0.277, *p* = 0.024), even when VAT was made relative to total trunk CSA (*r*^2^ = 0.303, *p* = 0.016) and TLM (*r*^2^ = 0.286, *p* = 0.021) (Table 5). Within this model, individuals with lower VAT_CSA_ and higher testosterone levels had higher Complex III activity. VAT + testosterone (*r*^2^ = 0.264, *p* = 0.029) and VAT + CRP (*r*^2^ = 0.286, *p* = 0.048) remained significant when controlling for TSI, even when VAT was made relative to total trunk CSA and TLM (Table 6). These relationships remained significant when controlling for age, with the exception of VAT + CRP (*r*^2^ = 0.264, *p* = 0.063) and VAT:TLM + CRP (*r*^2^ = 0.279, *p* = 0.053) which trended toward significance (Table 6). When controlling for LOI, VAT + CRP (*r*^2^ = 0.371, *p* = 0.015) remained significant even when VAT was made relative to total trunk CSA (*r*^2^ = 0.379, *p* = 0.014) and TLM (*r*^2^ = 0.372, *p* = 0.015). VAT + testosterone (*r*^2^ = 0.392, *p* = 0.003) also remained significant when controlling for LOI, even when VAT was made relative to total trunk CSA (*r*^2^ = 0.412, *p* = 0.002) and TLM (*r*^2^ = 0.397, *p* = 0.003) (Table 6). Again, individuals with lower VAT_CSA_ and higher testosterone levels had higher Complex III activity within this model. All other possible models to predict Complex III activity were tested and insignificant.

## Discussion

### Major Findings

This study determined mitochondrial health by CS and Complex III activity, surrogate markers for mitochondrial mass, and electron transport chain activity, respectively (O’Brien et al., 2017b). The current findings suggest that reduced mitochondrial mass and enzyme activity can be predicted by increased visceral adiposity, inflammatory signaling, and reduced testosterone levels. Research has demonstrated that inflammation, markers of anabolism, and visceral adiposity are independently associated with mitochondria dysfunction, yet how these factors interact to predict mitochondrial function in chronic SCI is unclear. The results of the current study suggest that, in individuals with chronic SCI, lower VAT_CSA_ and higher testosterone levels or lower VAT_CSA_ and lower CRP levels positively predict mitochondrial function. The goal of the current study was to elucidate these relationships further to inform future longitudinal intervention programs aimed at enhancing mitochondrial function. The hypothesis that a combination of increased VAT and inflammation along with reduced testosterone levels are associated with the mitochondrial dysfunction seen in chronic SCI was confirmed. To our knowledge, this is the first report to examine the combination of these variables on mitochondrial dysfunction in chronic SCI. Based on the current results, an intervention that reduces visceral adiposity, inflammatory signaling, and optimizes testosterone levels may improve mitochondrial health. Prior work demonstrated that neuromuscular electrical stimulation resistance exercise decreases VAT and inflammatory biomarkers while enhancing citrate synthase and succinate dehydrogenase activities in persons with SCI (Gorgey et al., 2019b,^2020^). Future work may examine the effects of exercise or pharmaceutical interventions on the VAT-inflammation-mitochondria axis.

### Mitochondrial Function and Visceral Adipose Tissue

VAT mass was negatively related to both CS and Complex III activity, which agrees with previous results (O’Brien et al., 2017b). The relationships between VAT and CS activity remained significant when controlling for TSI, age, and LOI. VAT was also negatively associated with Complex III activity when VAT was made relative to LM. VAT and VAT:total trunk CSA trended toward significance with Complex III and became significant when controlling for LOI, which may be due to VAT volume being associated with LOI (Farkas et al., 2018). Unlike previous results, VAT:SAT ratio was not independently associated with either CS or Complex III activity (O’Brien et al., 2017b). However, unlike the current study, these previous results were found only in men with motor complete SCI. Overall, there was a clear relationship between visceral adiposity and mitochondrial health. Individuals with lower VAT had higher mitochondrial mass and enzyme activity, which is in agreement with previous research (O’Brien et al., 2017b). Previous research has clearly shown that excess accumulation of VAT appears to play a significant detrimental role in cardiometabolic health (Freedland, 2004; Emmons et al., 2011; Gorgey and Gater, 2011). Indeed, O’Brien et al. (2017) demonstrated that lipid and metabolic profiles are related to mitochondrial mass and activity in individuals with SCI. Similar to the current results, O’Brien et al. (2017b) showed that many body composition measures remained related to CS and Complex III activity when normalized to thigh muscle CSA. Together, these results suggest that increased visceral adiposity results in decreased skeletal muscle mitochondrial mass and activity in individuals with chronic SCI. Future interventions targeting both reductions in VAT mass and improved mitochondrial function may enhance cardiometabolic health in individuals with chronic SCI.

### Mitochondrial Function and Testosterone

Testosterone was independently associated with Complex III and CS activity even when controlling for age and LOI. VAT + testosterone was also significantly associated with both Complex III and CS activity. Individuals with lower VAT_CSA_ and higher testosterone levels had higher CS and Complex III activity within these models. These relationships were robust and remained significant when VAT was made relative to total trunk CSA or LM. The levels in the current study were 346.72 ± 223.7 ng/dL, which included six females. When excluding the females, the levels were 428.08 ± 168.93 ng/dL. Six males (25%) in the current study were hypogonadal (<300 ng/dL), 11 males (46%) were in the low normal range (i.e., 301–500 ng/dL), and seven males (29%) were in the upper range of normal (>500 ng/dL). Therefore, the majority of the males in the current study were either hypogonadal or in the low normal range and could potentially benefit from therapeutic replacement. Men with SCI have reduced testosterone levels (Clark et al., 2008; Durga et al., 2011; Bauman et al., 2014; Sullivan et al., 2018), and reduced testosterone levels are associated with greater VAT mass in men with SCI (Abilmona et al., 2019). In persons with SCI, the decline in testosterone levels is 50% greater than in able-bodied individuals (Gray et al., 1991). VAT is inversely related to plasma total and free testosterone levels in healthy men (Seidell et al., 1990). Deficient testosterone levels increase cardiometabolic risk in young men with chronic SCI. Men with SCI that have total testosterone in the low normal range (i.e., 301–500 ng/dL) have increased risk compared to men with levels in the upper range of normal (>500 ng/dL) (Sullivan et al., 2018). Indeed, low serum testosterone in men with SCI is associated with a poorer cardiometabolic prognosis than men with high serum testosterone (Abilmona et al., 2019). Previous research showed that testosterone replacement therapy can reduce VAT (Gorgey et al., 2019b) and increase peroxisome proliferator-activated receptor-γ coactivator-1α (PGC-1α), the master regulator of mitochondrial biogenesis (Gorgey et al., 2020). Future interventions targeting both reductions in VAT mass and optimization of testosterone levels may improve cardiometabolic health in individuals with chronic SCI.

### Mitochondrial Function and Inflammation

CRP was significantly negatively associated with CS activity and trended toward a significant association with Complex III. VAT + CRP was also significantly negatively associated with CS activity. VAT + CRP trended toward a significant association with Complex III and became significant when VAT was made relative to total trunk CSA. Within these models, individuals with lower VAT_CSA_ and CRP levels had higher CS and Complex III activity. CRP is elevated in persons with chronic SCI (Gibson et al., 2008), is an important predictor of cardiovascular health, and can predict myocardial infarction and stroke (Ridker, 2003). In previous reports, CRP has been associated with VAT mass in various populations (Saijo et al., 2004; Pou et al., 2007; Faber et al., 2010). VAT surrounds the internal organs of the abdominal cavity (Després et al., 2001) and synthesizes and releases proinflammatory cytokines (Yudkin et al., 2005; Farkas and Gater, 2018). VAT secretes higher levels of IL-6, plasminogen activator inhibitor-1, and TNF-α compared to SAT (Farkas and Gater, 2018). While IL-6 and TNF-α were not independently associated with CS or Complex III activity in the current study, CRP is elevated in chronic SCI and is reportedly stimulated by both IL-6 and TNF-α (Edwards et al., 2008; Sam et al., 2009; Farkas and Gater, 2018). However, it is unknown if VAT directly contributes to elevated CRP levels in chronic SCI. Interestingly, IL-6 predicted mitochondrial health, but only in individuals over 40. IL-6 levels have been shown to increase with aging and are associated with increased cardiovascular disease risk (Rea et al., 2018). Future interventions targeting both reductions in VAT mass and inflammation may improve cardiometabolic health in individuals with chronic SCI.

## Limitations

It should be noted that this study does not precisely identify the complex causal relationships between inflammation, testosterone, anabolic markers, visceral adiposity, and mitochondrial health among those with chronic SCI. This is a correlative analysis based on baseline cross-sectional data. A small number of females (*n* = 6 out of 33, ∼18%) were included in these analyses; however, this may serve as an added benefit to the generalizability of the results of this study because this closely mirrors the actual proportion of males and females living with SCI (NSCISC Annual Report, 2020). This study used a small sample of healthy individuals (i.e., no cardiovascular disease, type 2 diabetes, pressure ulcers, or common medical and psychiatric comorbidities), limiting the generalizability of findings beyond individuals with similar levels of function. Another concern is that conducting simultaneous multiple regressions may result in a multicollinearity problem, especially with a small sample size.

It has been suggested that for a multiple regression with six or more predictors, a sample size of at least 100 is necessary (Green, 1991). In this study, constraints such as access to this specific population and the cost of MRI and DXA limited the sample size. We attempted to limit this multicollinearity problem by including a maximum of two predictors per model. Another limitation is that high variability within the sample resulted in non-normal distributions for several variables. Since we believe it is essential to capture the true variability within the chronic SCI population, we log transformed these variables rather than eliminating statistical outliers, which would have reduced our sample size. For example, removing outliers for VAT alone would have decreased our sample by ∼17% (*n* = 5). The results of this study are exploratory, evaluating potential predictors of mitochondrial function in chronic SCI. Considering these limitations, the current findings identify potential predictors of mitochondrial dysfunction following SCI. A large multi-center trial is highly warranted to address the aforementioned limitations effectively.

## Conclusion

Increased visceral adiposity, associated inflammatory signaling (CRP), and reduced testosterone levels predict mitochondrial dysfunction following SCI. Specifically, lower VAT_CSA_ and higher testosterone levels or lower VAT_CSA_ and lower CRP levels positively predict mitochondrial mass and enzyme activity in persons with chronic SCI (Figure 1). TNF-α, IGF-1, and IGFBP-3 were not related to mitochondrial function in this study. IL-6 predicted mitochondrial health, but only in individuals over 40. Future research should further investigate the causal relationships between visceral adiposity, inflammation, testosterone, and mitochondrial health in persons with chronic SCI. Furthermore, interventional studies should be designed to examine the efficacy of diet, exercise, and potentially testosterone replacement therapy on enhancing mitochondrial health in chronic SCI.

## Data Availability Statement

The raw data supporting the conclusions of this article will be made available by the authors, without undue reservation.

## Ethics Statement

The studies involving human participants were reviewed and approved by the McGuire Veteran Affairs Investigation Research Board and the Virginia Commonwealth University (VCU) Office of Research and Innovation approved the current study. The patients/participants provided their written informed consent to participate in this study.

## Author Contributions

RL, EL, and QC conducted the experiments. RG analyzed blood markers. JG analyzed the data, interpreted the experimental results, designed the figures and tables, and drafted the manuscript. AG, EL, and QC designed the study and interpreted the experimental results. RP assisted with the statistical modeling. AG established funding support. All authors reviewed, revised, and approved the final version of the manuscript.

## Conflict of Interest

The authors declare that the research was conducted in the absence of any commercial or financial relationships that could be construed as a potential conflict of interest.

## Publisher’s Note

All claims expressed in this article are solely those of the authors and do not necessarily represent those of their affiliated organizations, or those of the publisher, the editors and the reviewers. Any product that may be evaluated in this article, or claim that may be made by its manufacturer, is not guaranteed or endorsed by the publisher.

## Abbreviations

SCI: spinal cord injury
LOI: level of injury
MRI: magnetic resonance imaging
SAT: subcutaneous adipose tissue
VAT: visceral adipose tissue
VAT:SAT: ratio of VAT to SAT
LM: Lean mass
TLM: total lean mass
IL-6: interleukin-6
IGF-1: insulin-like growth factor 1
IGFBP-3: insulin-like growth factor binding protein 3
CRP: c-reactive protein
CS: citrate synthase
TNF-α: tumor necrosis factor alpha.
